# Football

**Published:** 1897-11

**Authors:** 


### FOOTBALL.

The death of young Gammon from injuries received in a recent football match has called forth legislative enactment which forbids the playing of the game for "prizes," and virtually puts an embargo upon an important branch of athletics.

The purposeless death of the young man in the full bloom of vigorous youth is greatly to be deplored, but it must be classed with fatalities occurring in boating, swimming, bicycle riding and other pastimes and does not furnish sufficient ground for abolishing the game of football any more than accidents in other sports render their compulsory discontinuance expedient.

Football, as it is now played, is unquestionably and unwarrantly brutal and should be modified to such an extent that injuries to the players would not be, as now, the rule,but the exception. The old free-to-all-helter-skelter game of a decade ago furnished just as much fun and wholesome exercise and, barring a barked shin and a bloody nose every now and then, was as harmless as "puss in the corner." Before adopting such drastic measures in the heat of resentment and protest, it would have been wiser to have "investigated" (what a charming word for the rural lawmakers) the game, and after a careful study strike out that which is dangerous, and shape the rules to the requirements of civilization and consideration for the integrity of the integumentary and osseous systems.

